# Workflow for the Implementation of Precision Genomics in Healthcare

**DOI:** 10.3389/fgene.2020.00619

**Published:** 2020-06-30

**Authors:** Sanja Mehandziska, Aleksandra Stajkovska, Margarita Stavrevska, Kristina Jakovleva, Marija Janevska, Rodney Rosalia, Ivan Kungulovski, Zan Mitrev, Goran Kungulovski

**Affiliations:** ^1^Zan Mitrev Clinic, Skopje, Macedonia; ^2^Bio Engineering LLC, Skopje, Macedonia

**Keywords:** exome, genome, personalized medicine, precision genomics, clinical practice, implementation

## Abstract

To enable the implementation of precise genomics in a local healthcare system, we devised a pipeline for filtering and reporting of relevant genetic information to healthy individuals based on exome or genome data. In our analytical pipeline, the first tier of filtering is variant-centric, and it is based on the selection of annotated pathogenic, protective, risk factor, and drug response variants, and their one-by-one detailed evaluation. This is followed by a second-tier gene-centric deconstruction and filtering of virtual gene lists associated with diseases, and VUS-centric filtering according to ACMG pathogenicity criteria and pre-defined deleteriousness criteria. By applying this filtering protocol, we were able to provide valuable insights regarding the carrier status, pharmacogenetic profile, actionable cardiovascular and cancer predispositions, and potentially pathogenic variants of unknown significance to our patients. Our experience demonstrates that genomic profiling can be implemented into routine healthcare and provide information of medical significance.

## Introduction

Personalized medicine is a proactive medical approach, which in general seeks to stratify patients in risk groups and tailor treatments, medical decisions, health promotion, or preventive measures according to the individual’s omics baseline profile combined with lifestyle and environmental factors (Ashley, 2016). The advent of cost-effective next-generation sequencing (NGS) technologies, such as the sequencing of whole genomes (WGS), whole and clinical exomes (WES and CES), combined with the accumulation of genetic knowledge and easy-to-use bioinformatics tools, has paved the way for genomics-based personalized medicine into clinics (Manolio et al., 2013; Goodwin et al., 2016; Doble et al., 2017; Vassy et al., 2017; Bylstra et al., 2019; Zoltick et al., 2019). Nowadays, these technologies have already started to transform healthcare by enabling precise disease screening, actionable diagnostics, treatment, and management. Despite this, precision genomics has not been fully implemented in the vast majority of healthcare systems yet. In order to facilitate its implementation, practical and user-friendly workflows and pipelines are required.

**FIGURE 1:**
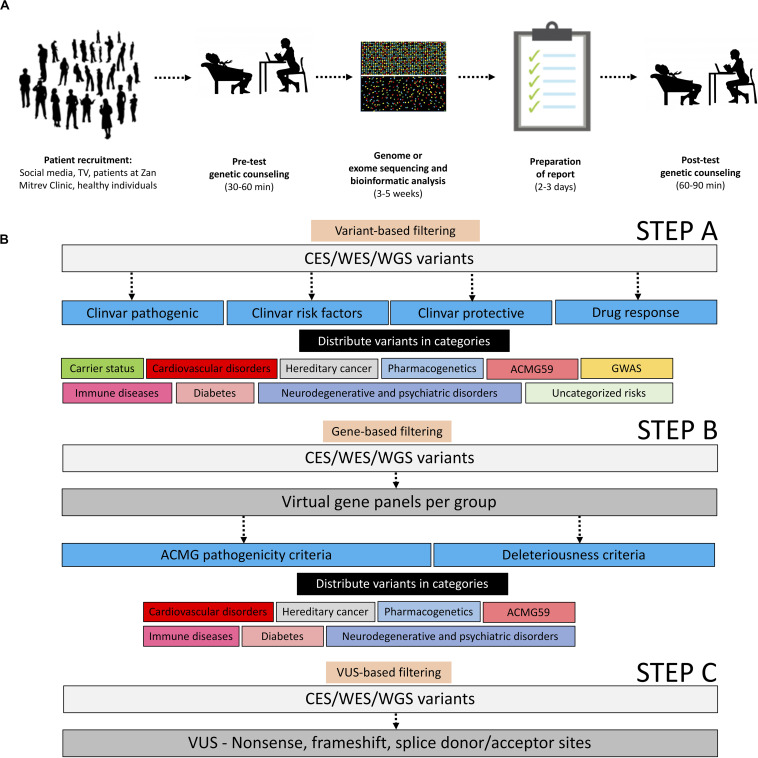
Operational and analytical workflows applied in this study. **(A)** Operational workflow starting with patient recruitment, pre-test genetic counseling, genome, or exome sequencing and bioinformatic analyses, representative sample of reports, and post-test genetic counseling. All adapted images used in this figure have a CC license. **(B)** Description of the analytical workflow for variant filtration.

In this study, we primarily aimed to describe a pipeline for balanced CES, WES, or WGS reporting of called variants in healthy individuals interested in proactive genetic testing. A batch of datasets taken from symptomatic patients was included only as a proof-of-principle. This approach of variant filtering helped us to initialize the process of implementing precision genomics in clinical practice at a tertiary healthcare institution (Figure 1). By applying this workflow, we were expecting to find actionable variants of clinical relevance or variants that might aid reproductive decisions. Our current experience demonstrates that the implementation of genomic profiling following our filtering pipeline into real-life clinical practice can provide information of medical significance. The pipeline could be used in future systematic and longitudinal studies focusing on the translational aspects of genomic medicine.

## Materials and Methods

### Patients

We evaluated 94 patients meeting our inclusion/exclusion criteria with a median age of 34.5 years (range 2 to 65) of which 51/94 (54%) were males and 43/94 (46%) were females (Table 1). All individuals/patients were recruited at the Zan Mitrev Clinic either through regular pro-active check-ups or TV/social media. The sole inclusion criterion for symptomatic patients was a referral from a medical specialist; patients who were not able to provide written informed consent and complete medical history were excluded from the study. In addition, healthy individuals who were unable to provide written informed consent were excluded from the study (Figure 1A). The analysis was done according to the workflow described in Figure 1B. The vast majority of patients were of Macedonian descent 74/94 (78.72%), followed by Albanian 11/94 (11.70%), Serbian 4/94 (4.25%), American 3/94 (3.19%), Turkish 1/94 (1.06%), and Bulgarian 1/94 (1.06%). The aforementioned protocol was used only as a proof-of-principle for analyzing genetic data from symptomatic patients (*n* = 15); we only communicated the mutations associated with the clinical phenotype. In contrast, full reports following this protocol were disclosed to all healthy individuals (*n* = 79). Symptomatic patients were informed that additional unrelated information concerning their carrier status and pharmacogenetic profile could be provided as well. Although the WGS analysis has advantages over WES and CES in the respect of providing more comprehensive and uniform coverage of the whole genome, most of the patients 89/94 (94.7%) underwent WES or CES testing, due to cost-effectiveness.

**TABLE 1:** Description of the cohort.

**Gender**	
Male	51 (54%)
Female	43 (46%)
**Healthy/Affected**	
Healthy	79 (84%)
Affected	15 (16%)
**Age**	
<18	9 (10%)
>18	85 (90%)
**Method**	
CES	29 (31%)
WES	60 (64%)
WGS	5 (5%)

### Ethics Statement

Written and signed informed consent for participation and publication of data was obtained from all subjects or their legal guardians (for patients under the age of eighteen) in this study. The ethics committee of the Zan Mitrev Clinic waived the need for IRB approval, deeming written and signed informed consent sufficient.

### DNA Extraction, Library Preparation, NGS Sequencing

Around 5 ml of whole blood was collected in K_2_-EDTA tubes, following accepted principles for blood drawing and blood collection. DNA was extracted from 400 μl of whole blood in a SaMag-12 automatic nucleic acid extraction system (Sacace Biotechnologies, Como, Italy), yielding between 5 and 15 μg of pure DNA, measured by NanoDrop spectrometry (A260/280 ratio 1.7–1.9). Clinical exome enrichment was carried out by using the TruSight One sequencing panel (Illumina, San Diego, United States) or in-house developed CES enrichment protocol (Sophia Genetics, Saint-Sulpice, Switzerland). Whole exome enrichment was carried out by using the SureSelect Human All Exon V6 kit (Agilent Technologies, Santa Clara, United States) or Human Core Exome kit (Twist Bioscience, San Francisco, United States). The entire wet lab work (DNA QC, enrichment, library preparation, and sequencing) for CES, WES, and WGS were carried out in the Sophia Genetics, Wuxi Nextcode or DNA link, or Beijing Genomics Institute facilities, respectively.

### Primary Bioinformatic Analysis

For CES, between 13 and 30 million reads were obtained with a NextSeq machine (Illumina, San Diego, United States), with a coverage of at least 50x for average 81% of all sequences. Sequence quality control was done with FastQC^1^, and sequences were mapped to hg19 with BWA (Li and Durbin, 2009). single nucleotide variant (SNV) and indel calling, together with advanced variant annotation, were done with the Sophia DDM platform (Sophia Genetics, Saint-Sulpice, Switzerland).

For WES and WGS, between 40 and 120 million reads or ∼950 million were obtained with a HiSeq X–10 machine or NovaSeq 6000 (Illumina, San Diego, United States), respectively. The coverage of WES or WGS was >75x or >40x, respectively. Alignment, variant calling, and annotation were done on the Genoox platform (Palo Alto, United States).

All CES, WES, and WGS variant lists were additionally annotated with Annovar, which provides more annotation notes than Sophia Genetics and Genoox (Yang and Wang, 2015). All detected variants were taken into consideration in the subsequent filtering steps.

### Secondary Bioinformatics Analysis, Filtering, and Interpretation of Variants

The list of high confidence annotated variants was downloaded directly from the Sophia DDM/Genoox platforms in a.txt or.csv format and analyzed further in a spreadsheet program such as Microsoft Excel. In the primary variant-based selection step the entire list of variants was filtered (either in Excel or in-platform) based on ClinVar terms “pathogenic,” “protective,” “risk factor,” and “drug response” followed by manual curation, manual filtration, and manual function attribution, and then distributed in the following categories: carrier status, cardiovascular disorders, hereditary cancer, pharmacogenetics, ACMG59 (Kalia et al., 2017), immune diseases, diabetes, neurodegenerative and psychiatric disorders, uncategorized risks, and genome-wide association studies (GWAS; MacArthur et al., 2017).

As a secondary gene-based filtering approach, by using the “virtual panel” capability within the Sophia DDM/Genoox platforms, we have created an array of standardized virtual gene panels encompassing genes associated with (1) cardiovascular disorders, (2) hereditary cancer, (3) neurodegenerative and psychiatric disorders, (4) diabetes, (5) immune diseases, and (6) ACMG59 genes (Kalia et al., 2017). The virtual gene lists per panel can be found in Supplementary File S1. Variants were selected from these virtual panels based on ACMG pathogenicity criteria (Richards et al., 2015) or computationally defined deleteriousness criteria (SIFT, Polyphen2, Mutation Taster, Mutation Assessor, FATHMM, dbscSNV Ada, GERP, GeneCanyon, and fitCons), and distributed in the above-mentioned categories.

Finally, in a third filtering variants of uncertain significance (VUS)-based approach, we selected all the non-sense/frameshift VUS in exons/splice donor-acceptor sites, and we reported them in the annex without further interpretation in order to make sure their significance is reassessed in the future when their function is determined (Figure 1B).

All selected variants were evaluated (selected or discarded) according to information in ClinVar and literature^2^. Also, further evaluation in other databases such as the human gene mutation database^3^, CentoMD database (Trujillano et al., 2017), and Clinvitae^4^ was carried out. Pathogenic variants, especially for medium and high penetrance alleles, were interpreted according to the latest available literature and ClinGen guidelines^5^. Disease risk for non-Mendelian, lower-penetrance variants associated with common diseases was assessed based on GWAS. Typically, the odds ratio or relative risk was reported, or in rare cases *P*-value or chi-square statistic, respectively.

To increase the detection rate and minimize the rate of false positives, first, we applied the entire protocol for all detected variants (low and high confidence), and then we did the same only for high confidence retained variants. Retained variants had a quality score >100, read depth >10, and quality by depth >10. To further reduce the level of false-positive variants, we retained only variants detected by multiple variant callers (GATK Haplotype Caller and FreeBayes). Alleles with a representation of >25% of the total read coverage were defined as heterozygous. All discrepancies were solved by additional manual evaluation.

### Patient Reports

Concerning the bioinformatics strategies for data analysis, filtering, and interpretation, we wanted to strike a balance between under- and over-reporting of variants; in other words, we wanted to maximize the benefit of the provided genetic analysis while reducing the costs and unnecessary follow-ups. Patient reports were divided into two sections, main report and annex, following the same line of reasoning as described previously (McLaughlin et al., 2014). The main report is typically 5–6 pages long and encompasses all the major findings (typically related to medium and high penetrance diseases), patient information, and methodology in a clear and concise language. In addition to the main report, we attached an annex consisting of 20–30 pages of all the selected (relevant) variants assigned to the above categories, in a tabular form accompanied with additional information such as:

• An in-depth reference to studies and major findings of the studies, especially for high penetrance alleles
• Odds ratio/relative risk/chi-square/*p*-value for common alleles (if available)
• Name of gene
• Type of variant (SNV, indel, etc.)
• Functional consequence (non-sense, missense, etc.)
• Genomic region (exonic, intronic, 5’UTR, etc.)
• Chromosome and chromosomal coordinates
• dbSNP or ClinVar rsID
• Population frequency (G1000, ExAC, esp5400)
• ClinVar signature (pathogenic, benign, drug response, etc.). This section reports the overall interpretation of a variation based on aggregating data from submitters.
• Inheritance (autosomal recessive, autosomal dominant, etc.)
• Level of evidence in the pharmacogenetics section (based on^6^ grading system)

### Genetic Counseling

All the patients underwent pre- and post-test genetic counseling. At the time of pre-test genetic counseling patients were informed about the potential implications of the genetic results to themselves and their families. We discuss the pros and cons of the test, and the current state of genetic/genomic research, as well as the basic principles of inheritance and penetrance. Following the test, we held in-depth discussions where the patients were reacquainted with the basics of DNA biology, genetic variants, types of inheritance, penetrance, and implications. With regard to common medical conditions and diseases, patients were told that these conditions are multifactorial and may include other known or unknown genetic, lifestyle, or environmental components. The genetic counselors stressed the meaning of phrases “no known pathogenic mutation causing/associated with [name of condition]” and the meaning and gravity of known pathogenic mutations.

Prior to testing, all of the patients signed written informed consent. All patients having actionable variants underwent a further examination or consultation with a relevant specialist. Hence, whenever mentioned further on that the patient was advised for a specific medical procedure, the advice came from relevant specialists and not the genetic counselors alone.

## Results

In our efforts to provide the most relevant genomic information to our patients, we divided variants into two categories: variants of known significance (VKS) obtained from the filtering steps A and B and VUS obtained from the filtering step C. Out of the VKS basket, we only selected variants with a defined clinical value (e.g., high penetrance, level 1a/1b pharmacogenetic association, direct involvement in a clinically relevant pathway). Out of the VUS basket, we reported only variants fulfilling ACMG pathogenicity criteria in medium and high penetrance disease-causing genes. For better reporting, every filtered variant was distributed in a fitting category (Figure 1B).

### Evaluation of Known Variants

#### Carrier Status of Rare Diseases

By selecting genetic variants annotated as “pathogenic” or “likely pathogenic” in the ClinVar database, supplemented by further manual curation, available literature evaluation, and filtration of low-frequency variants, the rare disease carrier status of each patient for known variants was derived. The vast majority of patients (96.2%) were carriers of at least one known rare disorder/condition with some of them carrying multiple pathogenic variants, median = 4 (Supplementary File S2).

#### Drug Response

Regarding “drug response,” the list of variants was interpreted using information from the pharmGKB^7^ database. Only level 1A and level 1B clinical annotations were added to the main report, while the rest of them remained in the annex. Most of the patients were carriers of multiple Level 1A/1B variants. For example, we observed that 34/94 (36.2%) patients were “poor/intermediate *CYP2D6* metabolizers,” which is relevant for the metabolism of many drugs, including anti-depressants, opioids, and tamoxifen. In order to reduce the misclassification rate of CYP2D6 metabolizers, we are currently implementing approaches for the detection of *CYP2D6* (and other genes) copy number variants from WES data. In addition, 23/94 (24.5%) patients were “poor/intermediate *CYP2C19* metabolizers” highly relevant for antiplatelet therapy with clopidogrel, in line with observations from our cohort of >3,000 patients (Klinceva et al., 2018). Similarly, 26/94 (27.7%) patients had a risk of statin-induced myopathy, with 2/26 (7.7%) being of a very high risk of myopathy and rhabdomyolysis as well (Supplementary File S2), in line with our internal observations from a cohort of >1,500 patients.

#### Actionable Variants Involved in Cardiovascular Diseases and Cancer

According to our protocol, we proceeded with the analysis of “actionable” variants (Table 2 and Supplementary File S2). Interestingly, more than one third of healthy individuals were carriers of pathogenic/potentially pathogenic variants leading to different types of arrhythmias and hereditary cancer, which are known to have incomplete penetrance.

**TABLE 2:** Representative list of patients with actionable variants.

Patient	Associated conditions with gene	Gene (ClinVar)	Nucleotide	Protein	rsID	Clinvar signature (interpretation)
2	Congenital long QT syndrome	*KCNJ2*	c.566G > T	p.Arg189Ile	rs199473381	Likely pathogenic(1)
12	Long QT syndrome	*KCNQ1*	c.914G > C	p.Trp305Ser	rs120074186	Pathogenic(2);Likely pathogenic(1)
29	Hereditary pancreatitis	*SPINK1*	c.101A > G	p.Asn34Ser	rs17107315	Risk factor(2);Pathogenic(4);Uncertain significance(3)
	Cystic fibrosis; Hereditary pancreatitis	*CFTR*	c.3154T > G	p.Phe1052Val	rs150212784	Likely pathogenic(3);Pathogenic(2);Uncertain significance(4);Drug-response(1)
30	Cardiac arrhythmia; Long QT syndrome	*ANK2*	c.11716C > T	p.Arg3906Trp	rs121912706	Likely benign(4);Pathogenic(2);Uncertain significance(2)
32	Malignant tumor of prostate; Hereditary cancer-predisposing syndrome	*MSR1*	c.877C > T	p.Arg293*	rs41341748	Pathogenic(1);Uncertain significance(3);Benign(1)
	Hereditary prostate cancer	*RNASEL*	c.793G > T	p.Glu265*	rs74315364	Pathogenic(1);Likely benign(1);Uncertain significance(1)
	Long QT syndrome	*KCNE1*	c.253G > A	p.Asp85Asn	rs1805128	Benign(5);Likely benign(5);risk factor(3);Pathogenic(1);Likely pathogenic(1);Uncertain significance(2)
43	Thrombophilia, Thyroid cancer	*HABP2*	c.1601G > A	p.Gly534Glu	rs7080536	Risk factor(2);Likely benign(1);Benign(1)
	Thrombophilia	*F5*	c.A1601G	p.Q534R	rs6025	Pathogenic(4);Risk factor(4);Benign(1)
52	Hereditary cancer risk	*CHEK2*	c.470T > C	p.Ile157Thr	rs17879961	Likely pathogenic(8);Pathogenic(9);Risk factor(3);Uncertain significance(2)
	MYH-associated polyposis; Hereditary cancer-predisposing syndrome	*MUTYH*	c.1437_1439delGGA	p.Glu480del	rs587778541	Pathogenic(14)
56	Prothrombin deficiency, congenital; Thrombophilia	*F2*	c.*97G > A		rs1799963	Pathogenic(4);Risk factor(4)
	Thrombophilia	*F5*	c.A1601G	p.Q534R	rs6025	Pathogenic(4);Risk factor(4);Benign(1)
81	Hereditary cancer-predisposing syndrome	*RAD50*	c.2801del	p.Asn934fs	rs748536322	Pathogenic(1)
84	Hereditary cancer-predisposing syndrome	*NBN*	c.511A > G	p.Ile171Val	rs61754966	Benign(3);Likely benign(1);Uncertain significance(11);Pathogenic(1);Risk factor(1)
91	Familial adenomatous polyposis	*APC*	c.3920T > A	p.Ile1307Lys	rs1801155	Likely benign(1);Likely pathogenic(3);Pathogenic(1);Uncertain significance(10);Risk factor(9)
92	Hereditary cancer risk	*CHEK2*	c.470T > C	p.Ile157Thr	rs17879961	Likely pathogenic(8);Pathogenic(9);Risk factor(3);Uncertain significance(2)
94	Prothrombin deficiency, congenital; Thrombophilia	*F2*	c.*97G > A		rs1799963	Pathogenic(4);Risk factor(4)

*The full table is given in Supplementary File S2. The asterisk denotes the variant in Clinvar https://www.ncbi.nlm.nih.gov/clinvar/variation/13310/.*

For instance, patient 2 was a carrier of a pathogenic variant (c.566G > T, p.Arg189Ile; rs199473381) in the *KCNJ2* gene, which has been associated with congenital long QT syndrome (Goldenberg and Moss, 2008). Follow-up EKG revealed visible abnormalities in the heart rhythm and the patient underwent further diagnostics. Patient 20 harbored a rare pathogenic/likely pathogenic variant (c.839C > T, p.Ala280Val; rs72552291) in the *GPD1L* gene that has been shown to decrease inward SCN5A Na + current and cause Brugada syndrome (Pfahnl et al., 2007). The patient underwent a regular cardiac exam, and EKG showed no visible abnormalities; since the patient was taking lithium therapy (which could conceivably unmask Brugada syndrome), advice was given to discuss this with their clinical psychiatrist and cardiologist. Furthermore, in patient 32 we discovered a variant (c.253G > A, p.Asp85Asn, and rs1805128) in the *KCNE1* gene that has been reported to be associated with long QT syndrome (Paulussen et al., 2004). During the post-test genetic counseling, the patient disclosed that a member of their close family had passed away “due to complications from arrhythmia.” Other patients were also found to carry variants associated with long QT or other channelopathies; patient 12 and their parent carried a pathogenic variant (c.914G > C; p.Trp305Ser; rs120074186) in the *KCNQ1* gene, whilst in patient 30 we identified a potentially pathogenic variant (c.5434C > T; p.Arg1812Trp; rs121912706) in the *ANK2* gene associated with sudden death of the young (Methner et al., 2016). Finally, in patient 43 and patient 56 we found a combination of variants (Marburg I and *F5* Leiden, *F2* and *F5* Leiden, respectively) that might significantly increase the risk of thrombosis (Voorberg et al., 1994; Poort et al., 1996; Hoppe et al., 2005). All patients were advised to consult a specialist and conduct follow-up studies if deemed necessary.

In regard to cancer, in patient 52, we discovered the presence of variants (c.1437_1439delGGA, p.Glu480del, and rs587778541) in the *MUTYH* gene and (c.470T > C, p.Ile157Thr, and rs1787996) in the *CHEK2* gene. The same *CHEK2* mutation was detected in patient 92. The variant in *MUTYH* is pathogenic and leads to MUTYH-Associated Polyposis in a recessive manner. The presence of the variant in a heterozygous format might slightly (1.5 times) increase the risk of colorectal cancer (Nielsen et al., 1993). The *CHEK2* variant has been reported to increase the risk of different types of cancer 2–3 times (Han et al., 2013). The patient was advised to consult a specialist and discuss a screening protocol. Next, patient 84 harbored the variant (c.511A > G, p.Ile171Val, and rs61754966) in the *NBN* gene, which is a low penetrance risk factor for cancer development (Gao et al., 2013); the patient reported having a family history of breast and pancreatic cancer. Finally, we detected a potentially pathogenic variant (c.3920T > A, p.Ile1307Lys, and rs1801155) in the *APC* gene in patient 91 (Leshno et al., 2016), who is currently undergoing follow-up diagnostics. All patients were advised to consult a specialist and conduct follow-up studies if deemed necessary.

### Evaluation of Potentially Pathogenic Variants of Uncertain Significance (VUS)

By analyzing VUS with rare population frequency meeting ACMG pathogenicity criteria (Richards et al., 2015) we uncovered many VUS in medium- or high-penetrance genes (Supplementary File S2). For instance, patient 56 is a carrier of (c.2423A > G, p.Tyr808Cys; rs746368140) in the *TGFBR3* gene. The involvement of the TGF-beta pathway has been reported in pathologies such as familial thoracic aortic aneurysm and dissection (familial TAAD; Milewicz and Regalado, 1993). The father of patient 56 was diagnosed with a thoracic and abdominal aortic aneurysm and underwent valve-sparing root replacement (Tirone-David procedure). The patient was advised to follow regular cardiovascular check-ups. Patient 75 is a carrier of VUS (c.1755dupA, p.Glu586fs, and rs751465048) in the *MLH3* gene, which is part of the MMR machinery associated with Lynch syndrome (Peltomaki, 2003). The patient already had benign tumors removed from their breast and nose, in the past. The patient was advised to consult a specialist. Finally, patient 84 is a carrier of (c.3145G > A, p.Gly1049Ser, and rs778181932) in the *FBN1* gene, which could be possibly associated with TAAD; the patient reported a history of sudden death in their close family.

## Discussion

The central tenet of personalized medicine is proactive care of patients based on the combined information and insights provided by omics approaches, lifestyle, environmental factors, and family history. To aid the implementation of precision genomics locally into our hospital, we have outlined a workflow centered around filtering, stratification in groups, and interpretation of genetic variants that can be readily applied in any genetic lab. By applying these strategies of variant-centric, gene-centric, and VUS-centric filtering, we were able to peer into the genetic constitution of 94 patients and make initial assessments of their carrier status, pharmacogenetics profile, and genetic risk of developing rare and common disorders.

Our experience demonstrates that the implementation of genomic profiling into real-life clinical practice can provide molecular and physiological information of medical significance, although many challenges remain to be addressed (Carter and He, 2016). To begin, serious efforts should be made to improve the knowledge of physicians and raise awareness for patients and the general public about the benefits and pitfalls of pre-emptive genomic testing, especially in the context of the current genetic knowledge. Second, standardization and defined guiding principles are necessary for both the technical and interpretational side of genomics in medicine. A list of guidelines (benchmarks) should be set for the minimal quality and coverage of sequencing data. For instance, currently WES is the most cost-effective approach, but it is limited to the protein-coding regions of the genome; as WGS sequencing costs continue to plummet this will likely lead to a rise in the popularity of WGS, which generates more uniform coverage of both coding and non-coding regions of the genome, relevant for monogenic as well as polygenic disorders. In addition, standardized algorithms for variant calling in clinical settings should be recommended. Moreover, more standardized approaches for filtering and distillation of relevant information, especially methods for calculation of polygenic scores, as well as balanced reporting of valuable information and VUS, and support tools for clinical interpretation, should be designed (Carter and He, 2016). Third, our analytical workflow based on filtering and virtual gene panels is readily applicable but still has a lot of space for improvement. For example, the virtual gene lists should undergo a process of constant curation and improvements from experts in the relevant subspecialties in order to get better informed, non-redundant, and more optimal lists of genes. Another limitation is that our focused study did not provide insights in regard to the cost-effectiveness of genetic testing, as well as the perceived value by both physicians and patients in a controlled and systematic manner. In order to objectively quantify the value of proactive genetic testing, longitudinal follow-up approaches are necessary.

Finally, many complex diseases, such as diabetes, cancer, and some neurological, cardiovascular, and psychiatric disorders, likely involve a large number of different genes and environmental factors (Hindorff et al., 2009; Ashley et al., 2010; De La Vega and Bustamante, 2018; Torkamani et al., 2018). These caveats sometimes might lead to unnecessary follow-up diagnostic measures and wastefulness of resources. Currently, the greatest value of genomic approaches lies in the detection of lower frequency moderate to high penetrance variants, which are easier to interpret and are better characterized due to their more resonant effects (Doble et al., 2017).

In conclusion, by establishing a balanced filtering pipeline, we set the foundation for the integration of genomics in mainstream clinical practice. The valuable insights and experiences we have obtained can have a bearing in future systematic and longitudinal follow-up studies.

## Data Availability Statement

The datasets generated for this study can be found in Zenodo 10.5281/zenodo.3830498 and url: https://doi.org/10.5281/zenodo.3830498.

## Ethics Statement

Written and signed informed consent for participation and publication of data was obtained from all subjects or their legal guardians in this study. Written and signed informed consent for participation and publication of data was obtained from the legal guardians of all patients under the age of eighteen. The ethics committee of the Zan Mitrev Clinic waived the need for IRB approval, deeming written, and signed informed consent sufficient.

## Author Contributions

GK conceived and designed the study. GK, SM, AS, MS, KJ, and MJ analyzed and interpreted the data. SM and AS participated in the genetic counseling process. IK and ZM contributed to the recruitment of patients and contributed intellectually. GK and RR wrote the manuscript. All authors contributed to the improvement of the manuscript and read the final version of the manuscript.

## Conflict of Interest

GK, AS, MJ, and IK are currently employed by the company Bio Engineering LLC. SM, RR, and ZM are currently employed by the private Zan Mitrev Clinic. MS was formerly employed by the Zan Mitrev Clinic. KJ did an internship at the Zan Mitrev Clinic.

## Footnotes

1 https://www.bioinformatics.babraham.ac.uk/projects/fastqc/

2 http://clinvar.com/

3 http://www.hgmd.cf.ac.uk/

4 http://clinvitae.invitae.com/

5 https://www.clinicalgenome.org/

6 www.pharmgkb.org

7 https://www.pharmgkb.org/

## Supplementary Material

FILE S1 Contains lists of genes for each category of virtual panels.

FILE S2 Contains variant information related to patient information, methodology, rare diseases, drug-response, actionable conditions, and VUS.

## Abbreviations

ACMG59: incidental findings in 59 genes recommended by American College of Medical Genetics and Genomics
CES: clinical exome sequencing
GWAS: genome-wide association studies
indel: insertion or deletion
SNV: single nucleotide variant
VUS: variants of uncertain significance
WES: whole-exome sequencing
WGS: whole-genome sequencing.
